# Discovering the abnormalities and functional importance of ferroptosis-related molecules in cervical cancer

**DOI:** 10.7150/ijms.107133

**Published:** 2026-01-01

**Authors:** Yu Sun, Junhua Zhang, Lingyu Guo, Jiaxin Zhang, Qian Chen, Ting Zhang, Jiaqi Yang, Yuting Zhang, Qianwei Zhen, Shuqi Chi, Gaishuang Shang, Baoxia Cui, Yunlong Cui, Youming Zhang, Youzhong Zhang, Sai Han

**Affiliations:** 1 Department of Obstetrics and Gynecology, Qilu Hospital of Shandong University, Jinan, Shandong 250012, P. R. China.; 2 Department of Obstetrics and Gynecology, the Second Qilu Hospital of Shandong University, No. 247 Beiyuan Street, Tianqiao District, Jinan, 250033, P. R. China.; 3 Eastsea Pharma Co. LTD, Qingdao, Shandong 266400, P. R. China.; 4 State Key Laboratory of Microbial Technology, Shandong University, Qingdao, 266237, P. R. China.

**Keywords:** TCGA, ferroptosis, GCH1, tumor microenvironment, macrophage polarization

## Abstract

Ferroptosis is an iron-dependent nonapoptotic form of cell death that links iron, lipid, and glutathione levels to a variety of disease-related activities. However, the characteristics of ferroptosis in cervical carcinoma (CC) are poorly understood. We acquired raw data on CC cohorts from The Cancer Genome Atlas (TCGA) and the Gene Expression Omnibus (GEO) database. Key genes were identified using differential gene expression analysis and intersected for further immune infiltration, transcription regulation, gene set variation analysis (GSVA), gene set enrichment analysis (GSEA), and drug sensitivity analysis. We also used immunohistochemical (IHC) staining to confirm the expression of important genes in cervical cancer tissue and their prognostic relevance. Finally, gene silencing and cell coculture experiments were used to verify the biological functional mechanism and its role in the tumor microenvironment (TME). Through bioinformatics analysis, we discovered that GCH1 and H1.2 are key ferroptosis-related molecules in cervical cancer. GCH1 and H1.2 could act as useful prognostic markers in cervical cancer, and in addition to their connection with the tumor microenvironment, the possible transcriptional regulatory network, hallmark pathways and chemotherapy sensitivity were also clarified. IHC of the tissue microarray (TMA) and immunofluorescence spatial distance evaluation revealed that GCH1 was more highly expressed in cervical cancer tissue than in paracarcinoma tissue. For patients with cervical cancer, higher GCH1 expression corresponded to a lower M2 cell proportion and a higher M1/M2 ratio as well as a greater GCH1-M2 distance. Silencing GCH1 in SiHa cells blocked the cell cycle, promoted apoptosis, and inhibited the migration and invasion abilities of the cells, possibly through the inhibition of the phosphorylated PI3K/AKT/mTOR pathway. Coculture of the cells with macrophages revealed that the silencing of GCH1 led to decreased expression of tumor necrosis factor (TNF), a biomarker of M1 macrophages. In this study, we performed a thorough investigation of ferroptosis-related genes and identified the functional complexity of GCH1 during tumorigenesis in cervical cancer.

## 1. Introduction

Cervical cancer is the most acute and frequently diagnosed gynecologic malignancy in women. In accordance with the latest cancer research data given by the World Health Organization, there were approximately 600,000 new cases and 340,000 deaths from cervical cancer in 2022 1. In China, cervical cancer remains the most common gynecological malignancy, with the number of new cervical cancer cases in 2022 increasing by more than 40,000 compared with that in 2020 2. Cervical cancer can be treated with surgery, radiation, and chemotherapy; however, 15-20% of patients in the early stage and 70% of patients with locally advanced cervical cancer relapse after treatment 3. The therapeutic options for these patients are limited, and their prognosis is inadequate, with a 5-year survival probability of approximately 17% 4. As a result, novel therapeutic options for patients with late-stage, recurring, or metastatic cervical cancer are urgently needed.

Ferroptosis, a form of programmed cell death driven by iron-dependent lipid peroxidation, has been reported to be a target for tumorigenesis and drug resistance in various tumors 5-7. Its primary morphological features include shrinking of the mitochondria, accompanied by a decrease in the mitochondrial ridge and an increase in the density of the mitochondrial membrane. Ferroptosis manifests biochemically as glutathione peptide (GSH) exhaustion, reduced glutathione peroxidase 4 (GPX4) activity, elevated lipid peroxidation products, and reactive oxygen species buildup 8-10. Although ferroptosis participates in the process of progression from precancerous lesions to cervical cancer 11, the specific functions of ferroptosis-related targets and their roles in the TME have not yet been elucidated 12.

In this study, we collectively identified the ferroptosis-related molecules in cervical cancer and revealed their potential connection with survival outcomes, the TME, the transcriptional regulatory network, hallmark pathways and chemotherapy treatment. Furthermore, we investigated the functions of the key genes as well as their roles in cervical cancer progression in cell experiments.

## 2. Materials and Methods

### 2.1 Data download

As the world's largest cancer genetic information database to date, TCGA (https://portal.gdc.cancer.gov/) provides gene expression, miRNA expression, copy number variation, DNA methylation, SNP expression, and related data. The processed mRNA expression profiles of cervical carcinoma tissues were obtained from the TCGA dataset containing both normal tissues and cervical cancer tissues, and the samples were divided into a normal group (n = 3) and a cancer group (n = 306). The classification of this dataset has been widely applied in other studies 13-17. The fragments per kilobase of transcript per million mapped reads (FPKM)_unstrand data of the TCGA-CESC dataset were used for further analysis. Furthermore, the GSE63514, GSE7410 and GSE7803 parallel matrix files were obtained from the NCBI GEO database. These datasets were employed as independent validation cohorts to assess the expression profiles of key genes. Specifically, the GSE63514 dataset consists of 28 cervical cancer samples and 24 normal samples, the GSE7410 dataset includes 35 cervical cancer samples and 5 normal samples, and the GSE7803 dataset comprises 21 cervical cancer samples and 10 normal samples. Moreover, a gene set of 698 ferroptosis-associated genes was acquired from GeneCards (https://www.genecards.org/).

### 2.2 Ferroptosis Gene Set Quantification and Survival Analysis in Cervical Cancer

The expression scores of ferroptosis-associated genes in cervical cancer samples obtained from the TCGA were quantified using single-sample gene set enrichment analysis (ssGSEA). Following this quantification, the samples were stratified into high- and low-expression groups based on the median gene set score. To evaluate the prognostic significance of the ferroptosis-associated genes, a log-rank test was used to compare the survival outcomes between these defined groups. The association between ferroptosis gene expression and overall survival in patients with cervical cancer was further illustrated through Kaplan‒Meier (KM) survival plots. Cox regression with time-dependent covariates was also performed.

### 2.3 Analysis of differential gene expression

The R limma package was utilized to examine the gathered information regarding the molecular mechanism of cervical carcinoma, investigate the variations in molecular expression between cancer and normal samples, and identify genes that were differentially expressed and met the requirements of |logFC| > 0.585 and P value < 0.05. Additionally, heatmaps and volcano plots for genes with differential expression were created.

### 2.4 Functional classification of genes with variable expression

With the purpose of gathering information about the biological roles and signaling pathways associated with the genesis and progression of cervical carcinoma, Metascape (www.Metascape.org) was employed to annotate and visualize the genes that were differentially expressed using Kyoto Encyclopedia of Genes and Genomes (KEGG) and Gene Ontology (GO) analyses. Statistical significance was indicated by a min overlap > 3 and p < 0.01.

### 2.5 Random survival forest algorithm

A random survival forest algorithm for screening distinctive genes was established using the software program randomForestSRC, and prognosis-relevant genes were evaluated based on their level of relevance (nrep=1000 indicates 1000 Monte Carlo repetitions). The LASSO algorithm automatically achieves feature sparsity through L1 regularization, effectively screening biomarkers that contribute most to classification. The implementation specifically utilized the "glmnet" R package, selecting the optimal regularization parameter λ (lambda) via 10-fold cross-validation, where this parameter was determined by minimizing the cross-validation error (deviance). Features with nonzero coefficients were ultimately retained as biomarkers with diagnostic value. Ultimately, the distinctive genes were identified as those with relative relevance values greater than 0.2.

### 2.6 Analysis of immune cell infiltration

A popular technique for assessing different types of immune cells in microenvironments is CIBERSORT. Using support vector regression, it simplifies the immunocyte subtype expression matrices. Twenty-two different human hematopoietic cell phenotypes, including T cells, B cells, plasma cells, and myeloid fractions, can be identified using 547 biomarkers available in CIBERSORT. The patient data in this study were analyzed using the CIBERSORT algorithm, which was also utilized for Spearman correlation analysis between the level of gene expression and immune cell content and to estimate the relative proportions of 22 hematopoietic cell morphologies.

### 2.7 Transcription regulation of key genes

Cistrome DB is a reasonably extensive database that includes transcription factor, histone modification, and chromatin accessibility samples from 30,451 humans and 26,013 mice, making it useful for investigating ChIP-seq and DNase-seq data. In this study, the regulatory functions of transcription factors in the expression of important genes were investigated using Cistrome DB. The transcription start point was set to 10 kb, and the genome reference file was set to hg38. Cytoscape was used to visualize the data.

### 2.8 GSVA

GSVA is an unsupervised, nonparametric technique for estimating transcriptome gene set enrichment. By thoroughly scoring the gene sets of interest and evaluating the biological activities of the samples, GSVA translates changes at the gene level into modifications at the pathway level. To quantify potential changes in the biological activities of various samples, gene sets from the Molecular Signatures Database were obtained for this study. Each gene set was then assessed using a thorough evaluation by the GSVA algorithm.

### 2.9 GSEA

The study's patient population was split into two groups based on the expression levels (low and high expression) of important genes. GSEA was used to further examine the differences in signaling pathways between the two groups. The MsigDB database provided annotated gene sets that were utilized as background gene sets for differential expression analysis of subtype pathways. The gene sets that were found to be significantly enriched (i.e., with an adjusted p value of less than 0.05) were ranked based on the sum of their individual scores. The study of disease subtypes and other studies with substantial biological importance frequently makes use of GSEA.

### 2.10 Drug sensitivity analysis

The pRRophetic package in R was used to estimate the chemotherapeutic sensitivity of each tumor sample based on the largest pharmacogenomics database, Genomics of Drug Sensitivity in Cancer (GDSC) (https://www.cancerrxgene.org/). Regression analysis was used to determine the IC50 value of each particular chemotherapeutic treatment. To confirm the correctness of the regression and predictions, ten cross-validation tests were carried out using the GDSC training datasets. All parameters, including the removal of batch effects, were set at their default values, and the expression levels of the same gene were averaged.

### 2.11 IHC staining

In this investigation, TMA technology was employed. The research followed the principles instituted by the Declaration of Helsinki. Tissues from 122 patients with cervical cancer were included in the TMA. Among them, 40 individuals donated tissues that were both malignant and paracancerous, whereas the remaining 82 patients donated only cervical cancer tissue. Four-micron-thick slices were cut from each TMA receiver block for TMA. Afterward, the slides were treated with antibodies against H1.2 (1:2000, Abcam, Cambridge, UK, AB4086) and GCH1 (1:1200, Proteintech, Wuhan, China, 28501-1-AP). Staining was performed with Panoramic MIDI (3D HISTECH) scanning equipment. Immunohistochemical staining was evaluated using the histochemistry score (H-Score). The comprehensive assessment methods were previously described 18.

### 2.12 Immunofluorescence spatial distance analysis and the M-IF staining protocol

For mIF, an Opal 5-color kit (TSAPLus, Servicebio) was utilized. Paraffin-embedded sections were rehydrated after being dewaxed. Each slide was first extracted at 125 °C for three minutes. The mixture was then allowed to cool naturally before being placed in PBS (pH 7.4) and shaken three times for five minutes each on a decolorizing shaker. The mixture was then incubated for ten minutes in H_2_O_2_. The membranes were subjected to multiple washes and blocking buffer treatments. For thirty minutes, the main antibody—used as a T lymphocyte cell marker—CD8 (ab237709, Abcam, 1:5000—was incubated with the samples at room temperature. Following washing, the slides were incubated with a secondary antibody conjugated with HRP for 10 minutes at room temperature. Subsequently, primary stainings for GCH1 (1:200, 28501-1-AP, Proteintech), CD11c (45581T, CST, 1:3000, used as an M1 macrophage marker), and CD163 (ab182422, Abcam, 1:5000, used as an M2 macrophage marker) were performed in the same manner. A 1:500 dilution of the anti-goat (GB23204, Servicebio) secondary antibody was used. A MIDI (3D HISTECH) scanning device was also used to analyze the spatial distance of immunofluorescence.

### 2.13 Cell lines

The human cervical cancer cell lines HeLa, SiHa, CaSki and C33A were purchased from Shanghai Zhong Qiao Xin Zhou Biotechnology Co., Ltd. Cell lines were validated through STR profiling. The cell lines were cultured at 37 °C with 5% carbon dioxide. The HeLa cell line was grown in DMEM (Gibco, Thermo Fisher Scientific) supplemented with 10% fetal bovine serum (FBS) (Life Technologies, Carlsbad, CA). The SiHa and CaSki cell lines were grown in RPMI 1640 medium supplemented with 10% FBS (Life Technologies, Carlsbad, CA). The C33A cell line was subsequently grown in MEM supplemented with 10% FBS (Life Technologies, Carlsbad, CA).

### 2.14 siRNA transfection

siRNAs targeting GCH1 or scramble sequences were synthesized by GenePharma Co., Ltd. (Shanghai, China). siRNAs were transfected into cells with Lipofectamine 2000 (Invitrogen, Thermo Fisher Scientific, USA). The sequences of the siRNAs used in our study are listed in Supplementary Table S1.

### 2.15 RNA extraction and quantitative real-time PCR

The total RNA of the cells was extracted with a SPARKeasy RNA Rapid Extraction Kit (Sparkjade, AC0205-B), and the concentration of each RNA sample was tested. The RNA was reverse transcribed into cDNA with a reverse transcription kit. Then, quantitative real-time PCR was performed for 40 cycles to amplify the target gene fragment, which was detected with a SYBR Green qPCR kit (Toyobo, Osaka, Japan). All the experiments were conducted at least three times. Relative gene expression levels were analyzed by the 2^-ΔΔCt^ method. The PCR sequences are listed in Supplementary Table S1.

### 2.16 Total protein extraction and Western blot (WB)

Total protein was extracted from the cells with RIPA lysis buffer (Beyotime) supplemented with 1% PMSF, and the protein concentration was assessed with a BCA kit provided by Thermo Scientific. The protein samples were separated by SDS‒PAGE, with a total of 30 μg per well. The proteins were subsequently transferred onto polyvinylidene fluoride (PVDF) membranes (Merck, ISEQ00010) by electrophoresis, and the membranes were subjected to blocking with 5% nonfat milk solution at room temperature for a period of 2 h. The membranes were then subjected to overnight incubation at 4 °C with the primary antibody, which was appropriately diluted. Afterward, the membranes were rinsed with TBST and then incubated with suitable HRP-conjugated secondary antibodies for 1.5 h at room temperature. The band signals were detected using Immobilon Western Chemiluminescent HRP Substrate (Merck) and then quantified using ImageJ software. β-Actin was utilized as the endogenous control. Each experiment was repeated at least three times. The primary antibodies used were as follows: anti-p-PI3K (1:1000, Cell Signaling Technology, #4228T), anti-p-AKT (1:1000, Cell Signaling Technology, #4060S), anti-p-mTOR (1:1000, Cell Signaling Technology, #2971S), anti-GCH1 (1:3000, Proteintech, 28501-1-AP), and anti-β-actin (1:80000, ABclonal, #AC026).

### 2.17 Cell migration and invasion assays

The migratory capacity of the cells was assessed in a Transwell assay. A total of 6 × 10^4^ cells resuspended in 200 μL of serum-free medium were seeded into the upper chambers of culture inserts (Corning, 3422). A total volume of 600 μL of culture medium containing 20% FBS was subsequently added to the lower chambers. After 24-48 h of incubation at 37 °C, the cells on the lower surface of the Transwell were fixed in methanol for 15 min. Following fixation, these cells were stained with 0.1% crystal violet for 20 min. Subsequently, images of the cells were captured using a microscope. The process of evaluating invasion was similar to that for evaluating migration, but the quantity of cells seeded during invasion was as high as 12 × 10^4^. Additionally, a mixture consisting of a 60 μL mixture of Matrigel (BD Biosciences) and medium was added to the membrane. The duration of invasion was extended to 36 h.

### 2.18 CCK-8

A Cell Counting Kit-8 (CCK-8) assay kit (Zhongshan Golden Bridge, Beijing, China) was used to evaluate cell viability. Briefly, 10 μl of Cell Counting Kit solution was added to each well of the culture medium and incubated for 2 h. The absorbance was determined at a wavelength of 450 nm.

### 2.19 Flow cytometry

For the cell cycle assay, cells were harvested for transmembrane processing to facilitate propidium iodide (BD, 550825) labeling. The samples were detected with a CytoFLEX flow cytometry instrument manufactured by Beckman Coulter. The data were evaluated using the ModFit LT program. For the apoptosis assay, we used an Annexin V-FITC/PI Apoptosis Kit (Elabscience) to label apoptotic cells. The results were analyzed using CytExpert software.

### 2.20 Cell Cocultures

THP-1 cells (acute monocytic leukemia) were cultured in RPMI 1640 medium (Gibco, Thermo Fisher Scientific, Waltham, MA) supplemented with 10% FBS (Life Technologies, Carlsbad, CA) and 1% penicillin‒streptomycin (100×) (Solarbio, Beijing, China) at 37 °C with 5% CO_2_. THP-1-derived macrophages were collected after treatment with phorbol ester (PMA, Sigma‒Aldrich, St. Louis, MO) (50 ng/ml) for 48 h. Then, we collected conditioned medium (CM) from GCH1-knockdown SiHa cell cultures as well as the scramble group and cultured them with M0 THP-1 cells. Following a 48-h period of coculture, the macrophages were collected to measure the mRNA expression levels of M1/M2 markers.

### 2.21 Analytical statistics

In our bioinformatics sections (2.1-2.10), the statistical analysis was performed using the R programming language (4.0). In the experimental section, GraphPad Prism version 8.0 (GraphPad Software Inc., San Diego, CA, USA) was used for statistical analysis. The data are expressed as the means with standard deviations (SDs), and statistical comparisons were performed using a Student's t test or a chi-square test. P < 0.05 was considered to indicate a statistically significant result.

## 3. Results

### 3.1 Pathway enrichment and differentially expressed gene intersection

**Figure 1:**
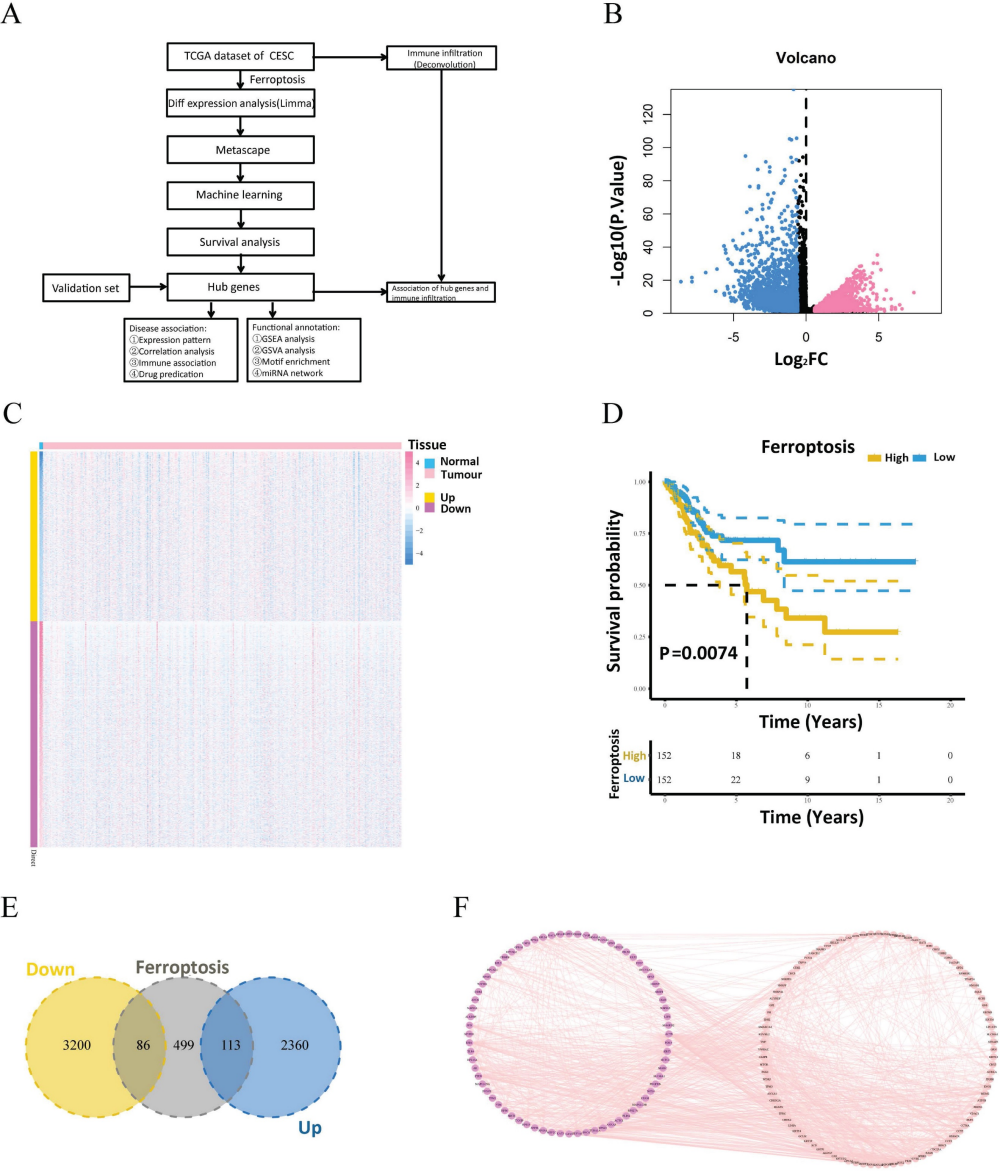
Workflow and expression of differentially expressed ferroptosis-related genes in cervical cancer. (A) Schematic diagram of the study workflow. (B) Volcano plot of differentially expressed genes between the tumor and normal groups. (C) Heatmap of the visualized gene network. The expression levels are shown in different colors, with purple indicating stronger expression and yellow indicating weaker expression. (D) Kaplan‒Meier survival analysis of patients with cervical cancer stratified by ferroptosis-related gene expression. (E) Venn diagram showing the intersecting genes between the differentially expressed genes and ferroptosis-related gene sets. (F) Protein interaction pairs associated with 199 differentially expressed ferroptosis-related genes from the STRING online database.

The processed mRNA expression data of 309 patients with cervical cancer were obtained from TCGA. Among these samples, 306 were tumorous, and 3 were normal. Fig. 1A presents a flow diagram summarizing the experimental design and bioinformatics analyses described in this article. The differentially expressed genes of the two groups were computed using the limma program. A total of 5759 genes, including 2473 upregulated and 3286 downregulated genes, satisfied |logFC| > 0.585 and a P value < 0.05 (Fig. 1B, C). Following the mapping of the differentially expressed genes into ferroptosis-related gene sets, 199 intersecting genes (of which 113 were upregulated and 86 were downregulated) were obtained (Fig. 1E). Next, the protein pairs of these 199 differentially expressed ferroptosis-related genes were obtained from the STRING online database and visualized using Cytoscape (Fig. 1F). Pathway analysis of these 199 differentially expressed ferroptosis-related genes revealed that the differentially expressed genes were enriched primarily in pathways related to ferroptosis, the regulation of DNA metabolic processes, the regulation of lipid metabolic processes, the regulation of cellular catabolic processes, and central carbon metabolism in cancer (Supplementary Fig. 1A and B).

### 3.2 Identification of important ferroptosis-related genes

**Figure 2:**
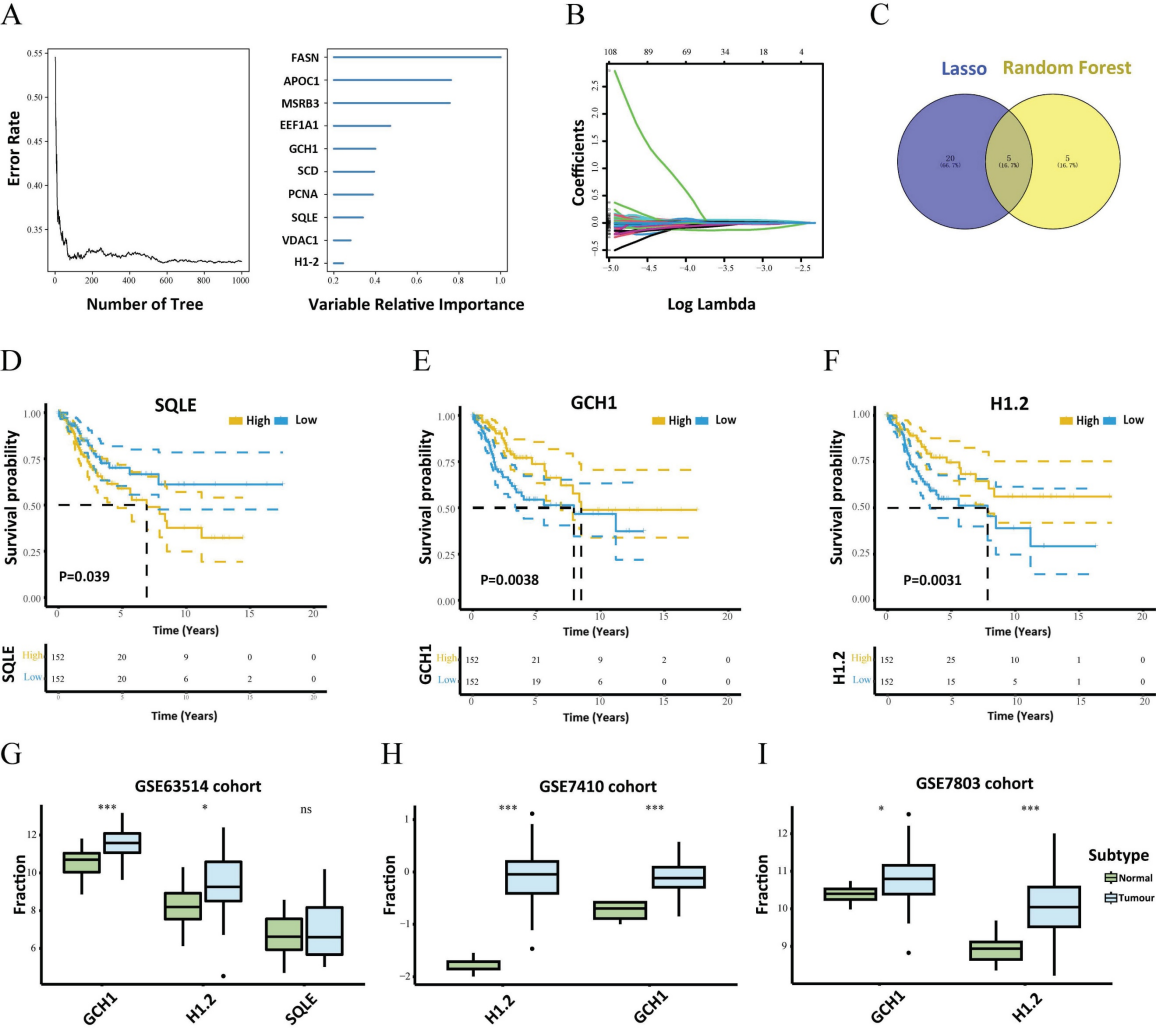
Search for key genes. (A) The random survival forest algorithm was used to screen the characteristic genes and rank the importance of 10 prognosis-related genes. (B) LASSO regression identified 25 genes as characteristic genes. (C) Venn diagram showing the number of genes common to and unique to the random survival forest analysis and LASSO regression. (D-F) Survival analysis of the key genes, including SQLE, GCH1 and H1.2. (G) Verification of SQLE, GCH1 and H1.2 from GEO. The expression levels of GCH1 and H1.2 were increased significantly in the tumor groups. (H-I) Differential expression of GCH1 and H1.2 in the external validation sets GSE7410 (H) and GSE7803 (I) in the tumor and normal groups.

To identify the major differentially expressed ferroptosis-related genes, a random survival forest analysis of these 199 genes was performed, and genes whose relative relevance levels were greater than 0.2 were chosen as the final markers. Figure 2A depicts the order of importance of the ten genes. Furthermore, LASSO regression was used to screen for characteristic genes, and 25 genes, which were subsequently identified as important genes, were selected as cervical carcinoma-specific genes (Fig. 2B). Five genes intersected between the random survival forest analysis results and the LASSO regression results (Fig. 2C). The genes SQLE, APOC1, H1.2, GCH1, and EEF1A1 were determined to be associated with cervical cancer. Furthermore, survival analysis of these five genes revealed that GCH1, H1.2, and SQLE expression predicted substantial variations in survival (Fig. 2D-F). The GEO GSE63514 dataset of patients with cervical cancer was downloaded to validate these three genes. The expression profiles of GCH1 and H1.2 in the GSE63514 dataset were compatible with those in the TCGA dataset (p < 0.05). Cox regression with time-dependent covariates of GCH1 and H1.2 was also significant (Supplementary Fig.2). As a result, GCH1 and H1.2 were selected as important genes for further study (Fig. 2G). The differential expression of the key genes GCH1 and H1-2 between cervical cancer tissues and normal cervical tissues was further substantiated using the GSE7410 and GSE7803 datasets (Fig. 2H-I).

### 3.3 Links between important genes and the TME

**Figure 3:**
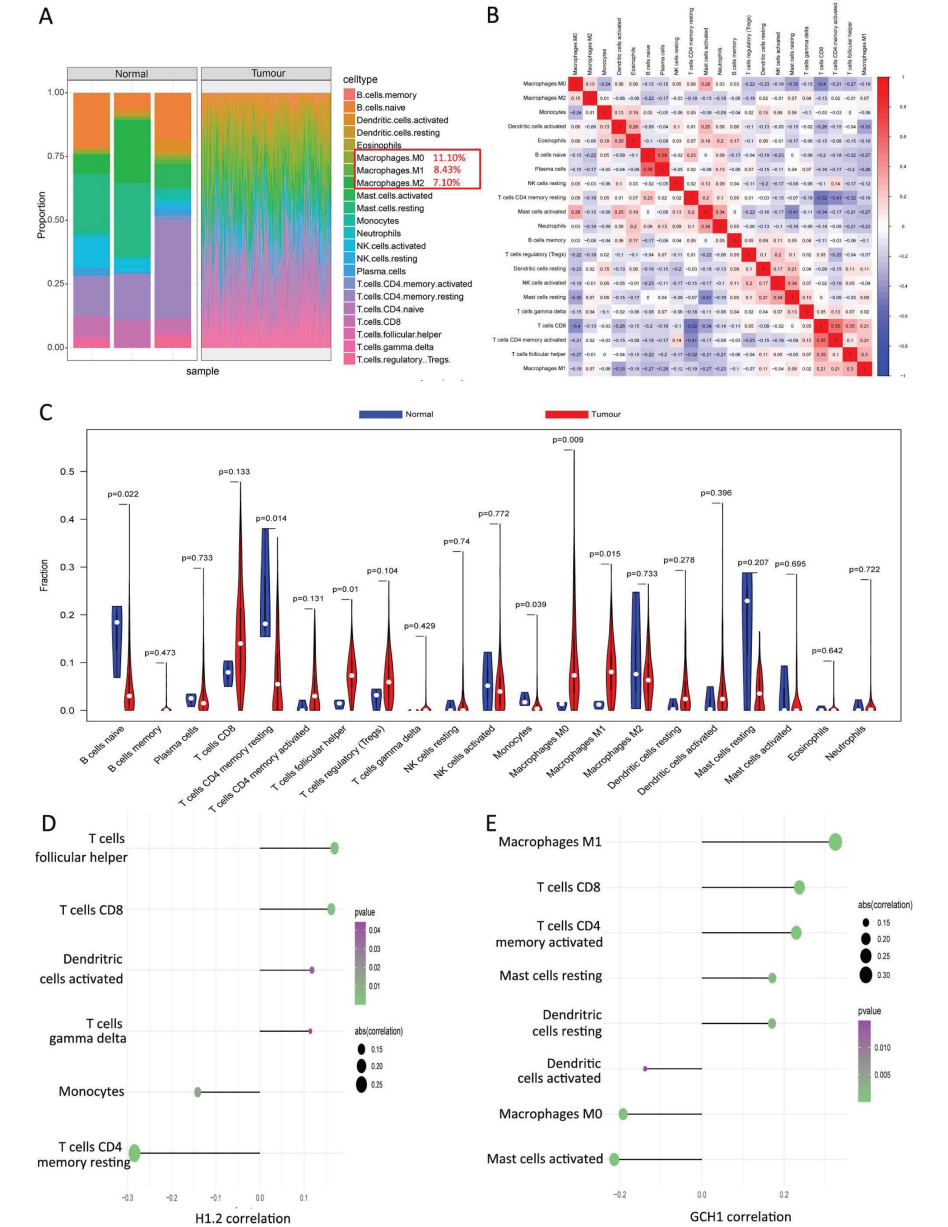
Relationships between the key genes and immune infiltration. (A) The immune cell contents of the normal and tumor groups. (B) Immune cell correlation map. (C) Comparison of immune cells between the tumor and normal groups. (D, E) Correlations between CIBERSORT and the expression levels of H1.2 and GCH1.

The TME is composed of fibroblasts, immune cells, extracellular matrices, numerous growth hormones, inflammatory agents, and unique physical and chemical features that influence disease diagnosis, survival rates, and clinical therapy sensitivity. The links between important genes in tumor datasets and immune infiltration were investigated to determine how key genes influence the development of cervical cancer. Figure 3A and Supplementary Table S2 shows the immune cell composition of the patients. Many significant correlations were discovered between immune infiltration levels (Fig. 3B). Compared with the normal group, the cancer group presented considerably greater numbers of follicular helper T cells, M0 macrophages, and M1 macrophages (Fig. 3C). Further investigation into the links between essential genes and immune cells revealed that the two critical genes (H1.2 and GCH1) were strongly associated with immune cells. H1.2, in particular, was positively correlated with follicular helper T cells and CD8+ T cells but negatively correlated with resting memory CD4+ T cells and monocytes (Fig. 3D). GCH1 was positively correlated with M1 macrophages and CD8+ T cells but negatively correlated with activated mast cells and M0 macrophages (Fig. 3E). Supplementary Figure 3 displays the findings of the correlation study between the two major genes, macrophages, and immunological checkpoints. These findings indicate that the two main genes were strongly associated with immunocyte infiltration levels and played important roles in the tumor immune microenvironment.

### 3.4 Transcriptional regulatory network of the two key genes

The transcriptional regulatory network of the two important genes, H1.2 and GCH1, was investigated. First, CistromeDB was utilized to predict the transcription factors for the two essential genes. GCH1 and H1.2 each had an estimated 94 transcription factors. The transcription factors were then displayed using Cytoscape, which revealed a comprehensive transcriptional regulatory network of essential genes for cervical cancer (Supplementary Fig. 4A). Additionally, the Microcode database was used to backward-predict the two important genes, resulting in the acquisition of 35 miRNAs and 37 mRNA‒miRNA pairs. These data were viewed in Cytoscape (see Supplementary Fig. 4B).

### 3.5 Potential GCH1 and H1.2 signaling pathways affecting tumor progression

**Figure 4:**
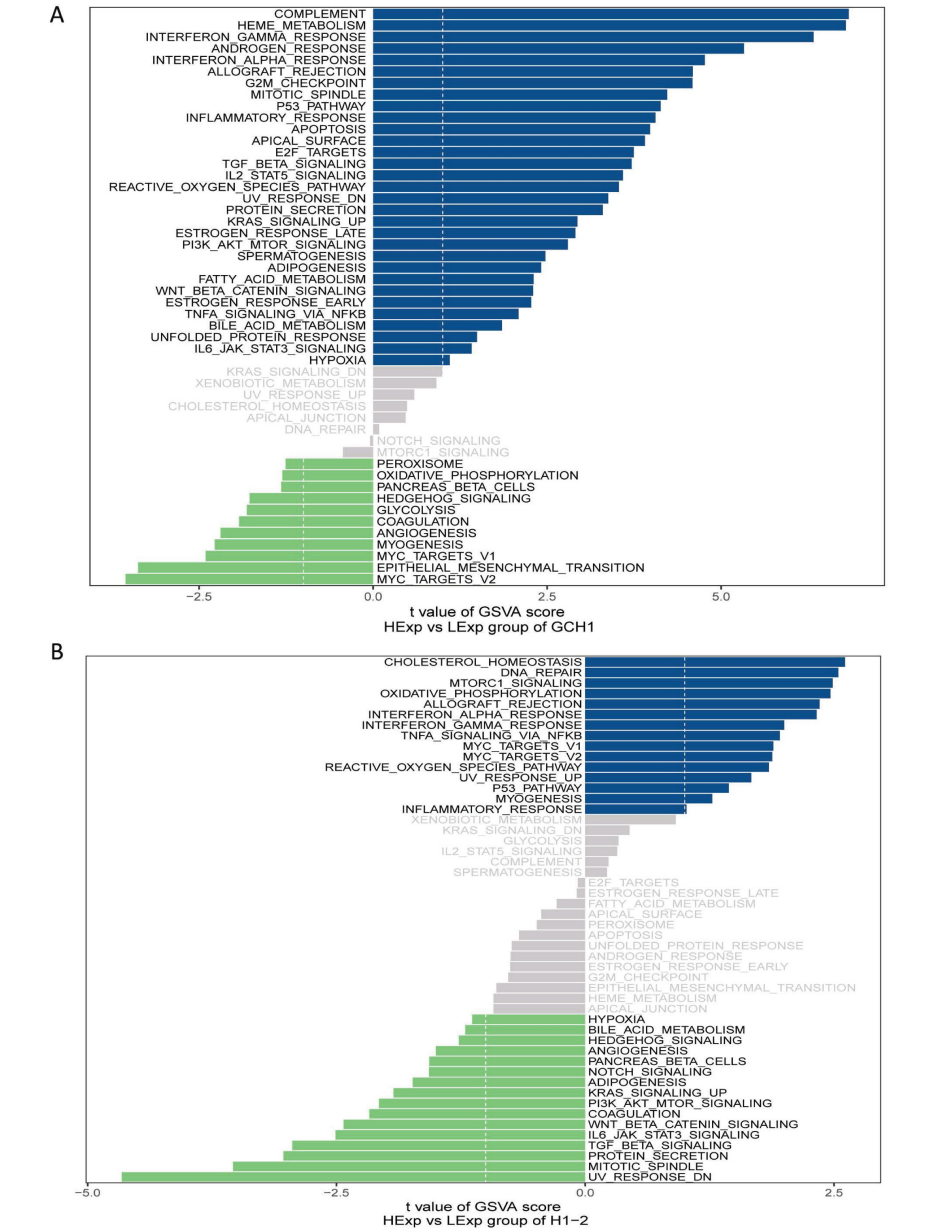
GSVA analysis of the key genes. (A) Enriched signaling pathways associated with highly expressed GCH1. (B) Enriched signaling pathways associated with highly expressed H1.2.

Next, the critical genes' signaling pathways, as well as the putative molecular processes by which GCH1 and H1.2 promote tumor growth, were investigated. The GSVA results revealed that highly expressed GCH1 was enriched in the COMPLEMENT, HEME_METABOLISM, and INTERFERON_GAMMA_RESPONSE signaling pathways (Fig. 4A). H1.2 was highly expressed in the CHOLESTEROL_HOMEOSTASIS, DNA_REPAIR, and MTORC1_SIGNALING signaling pathways (Fig. 4B).

### 3.6 GSEA of GCH1 and H1.2

**Figure 5:**
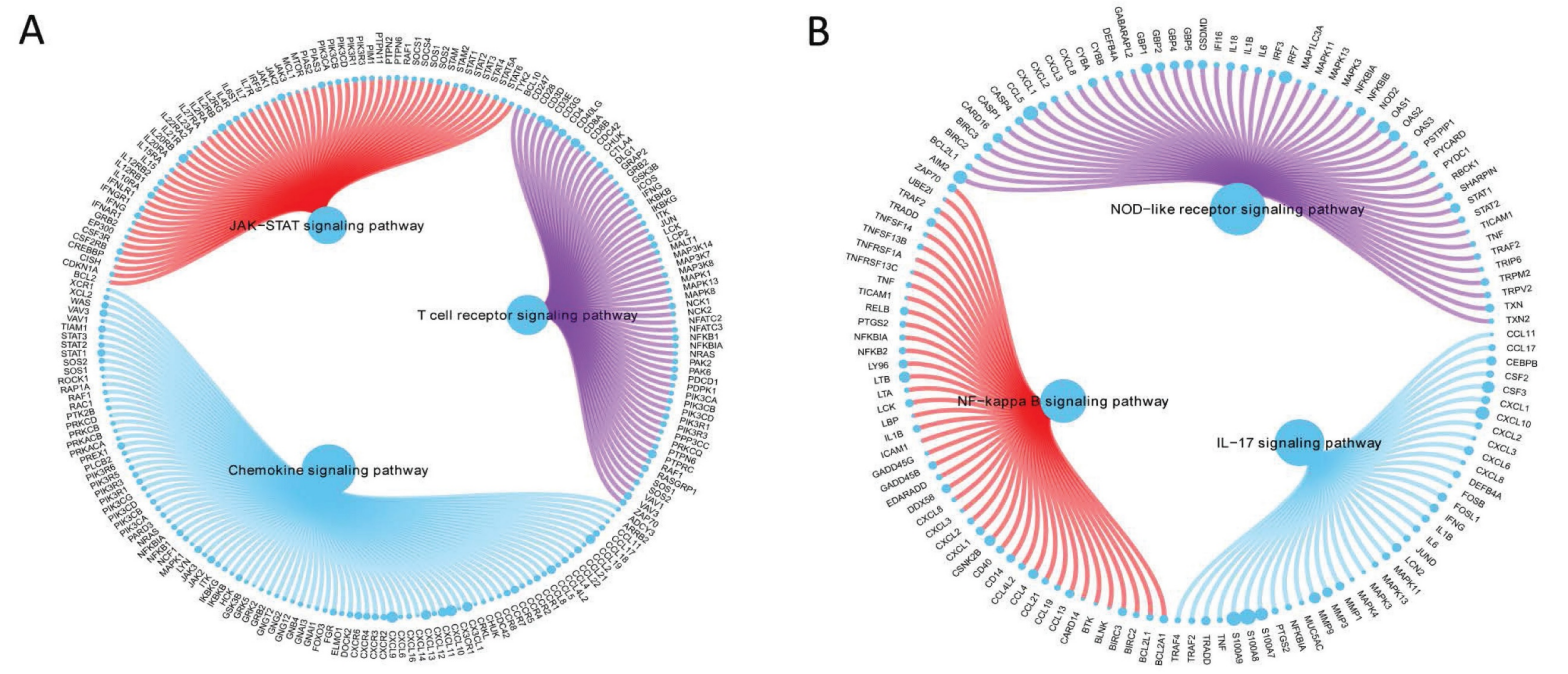
GSEA of GCH1 (A) and H1.2 (B).

GCH1 expression increased the activity levels of the chemokine, JAK/STAT, and T-cell receptor signaling pathways (Fig. 5A), whereas H1.2 expression increased the activity levels of the IL-17, NF-κB, and NOD-like receptor signaling pathways (Fig. 5B).

### 3.7 Possible sensitive chemotherapeutic medicines for GCH1 and H1.2 by the GDSC

Surgery combined with chemotherapy has been shown to be effective in curing early cervical cancer. In this study, the chemotherapy sensitivity of each cervical cancer sample was evaluated using the pRRophetic package in R based on drug sensitivity data from the GDSC, and patient sensitivities to common treatments and connections with GCH1 and H1.2 expression were investigated further. GCH1 expression was associated with patient sensitivity to A.770041, GSK269962A, KU.55933, BAY.61.3606, and CHIR.99021 (Supplementary Fig. 5A), and H1.2 expression was associated with patient sensitivity to A.770041, BMS.754807, BAY.61.3606, and CHIR.99021 (Supplementary Fig. 5B).

### 3.8 Relationships of carcinoma-related genes with GCH1 and H1.2

GeneCards (https://www.genecards.org/) provided information on the cervical carcinoma-related genes. The expression profiles of the two important genes and the top 20 cervical carcinoma-related genes with the greatest relevance scores were compared between the groups. The normal and cancer groups presented significantly different expression levels of BRCA1, BRCA2, BRIP1, CDH1, CDKN2A, CHEK2, ERBB2, MSH2, PALB2, PTEN, and TP53 (Supplementary Fig. 6A). We subsequently performed a correlation analysis between the key genes and cervical carcinoma-related genes and discovered substantial associations between the expression levels of the two key genes and the expression of several cervical carcinoma-related genes. GCH1 had a substantial positive correlation with PALB2 (r = 0.31), whereas H1.2 had a significant negative correlation with APC (r = -0.21) (Supplementary Fig. 6B).

### 3.9 Verification of GCH1 and H1.2 expression by IHC

**Figure 6:**
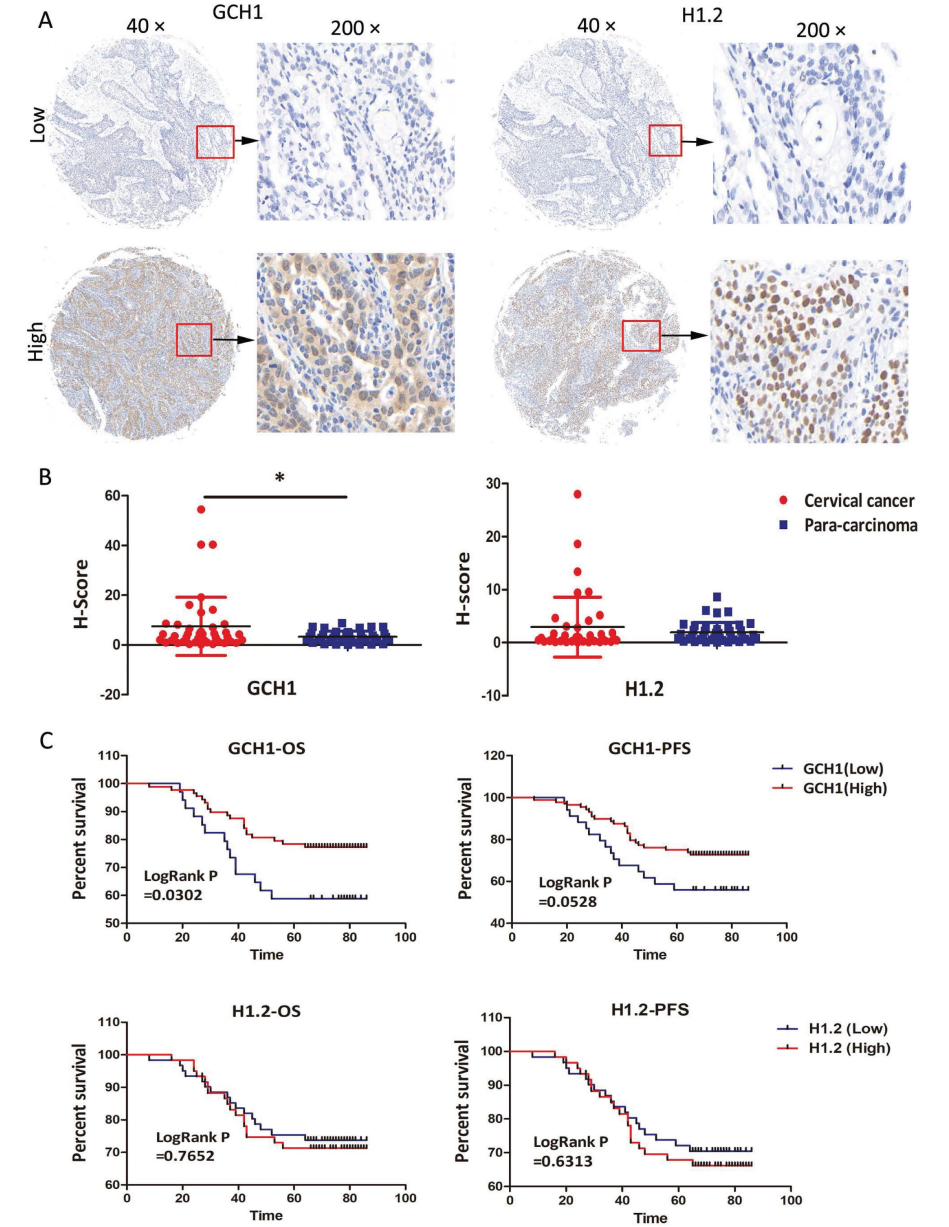
Immunohistochemical verification of GCH1 and H1.2. (A) Localization of GCH1 and H1.2 in cervical cancer tissues (×40 and ×200). (B) Expression levels of GCH1 and H1.2 in cervical cancer tissue and paracarcinoma tissue. (C) OS/PFS of patients with cervical cancer with high GCH1/H1.2 expression compared with those with low GCH1/H1.2 expression.

The expression profiles of GCH1 and H1.2 were confirmed in the TMA using IHC. The samples were sorted into two groups based on staining intensity and H-scores for GCH1 and H1.2 (Fig. 6A). GCH1 was expressed in both the cytoplasm and the nucleus, whereas H1.2 was expressed in only the nucleus (Fig. 6A). GCH1 expression was much higher in cervical cancer tissue than in paracarcinoma tissue (P < 0.05). There was no significant change in H1.2 expression between cervical cancer and paracarcinoma tissues (Fig. 6B). Survival analysis revealed a significant correlation between GCH1 expression and overall survival time (P < 0.05). Interestingly, the stronger the expression of GCH1 in cancer tissue was, the better the prognosis of these patients with cervical cancer (Fig. 6C). GCH1 expression levels were correlated with the cervical cancer FIGO clinical stage (P < 0.05) but not with age, human papilloma virus (HPV) infection, lymph node metastasis, histological grade, or cancer recurrence (Table 1). However, H1.2 expression levels did not correspond with either of these clinicopathological variables (Supplementary Table 3).

### 3.10 Immunofluorescence spatial distance analysis of GCH1

**Figure 7:**
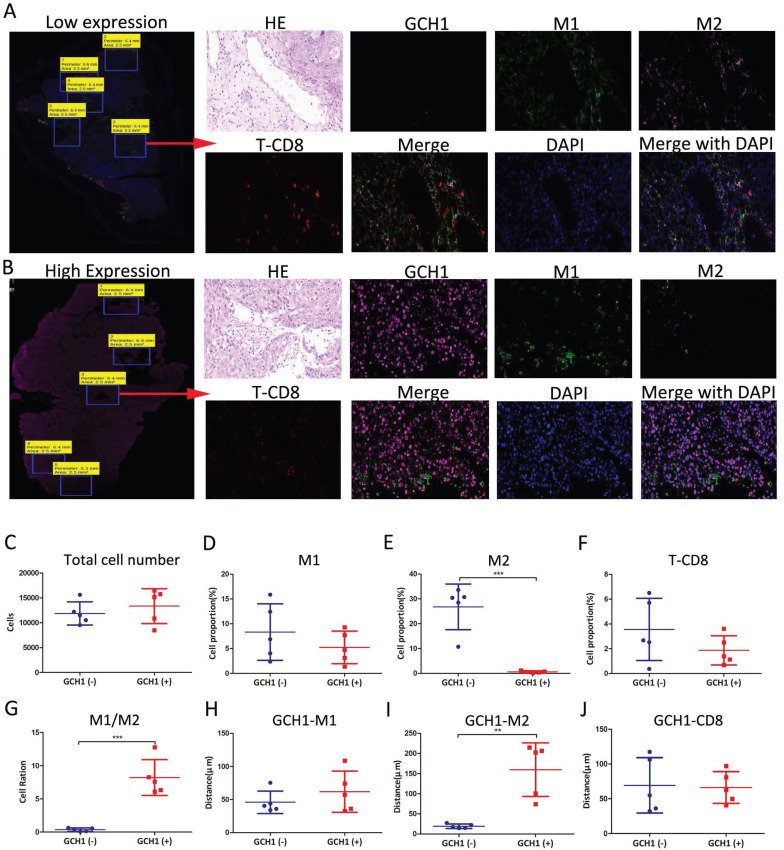
Immunofluorescence spatial distance analysis of GCH1. (A and B) Expression and localization of GCH1, CD11c, CD163 and CD8 in cervical cancer tissues. (C) Total cell numbers in Low GCH1 and High GCH1 cervical cancer tissues. (D-F) CD11c+ M1, CD163+ M2 and CD8+ T immune cell proportions in Low GCH1 and High GCH1 cervical cancer tissues. (G) M1/M2 cell ratios in Low GCH1 and High GCH1 cervical cancer tissues. (H-J) Distances among CD11c+ M1, CD163+ M2 and CD8+ T immune cells and tumor cells in Low GCH1 and High GCH1 cervical cancer tissues.

We performed immunofluorescence spatial distance analysis to determine the quantity and location of three immunological targets in cervical cancer tissue. We further split the histopathological sections into two groups based on GCH1 expression and evaluated the differences in immune infiltration (Fig. 7A, B and Supplementary Fig. 7).

In the Low GCH1 group, there were 11,862 cells, whereas in the High GCH1 group, there were 13,346 cells (P = 0.45) (Fig. 7C). In the Low GCH1 group, the proportion of CD11c+ M1 macrophages was 8.33%, whereas in the High GCH1 group, it was 5.24% (P = 0.32). In the Low GCH1 group, the percentage of CD163+ M2 macrophages was 26.79%, whereas in the High GCH1 group, it was 0.63% (P = 0.0002). The fraction of CD8+ T lymphocytes was 3.56% in the Low GCH1 group and 1.87% in the High GCH1 group (Low GCH1 versus High GCH1, P = 0.21) (Fig. 7D-G). The average distance between GCH1-CD163 in tumor cells and immune cells significantly differed between the Low GCH1 and High GCH1 groups (19.24 μm vs. 159.80 μm, P = 0.0015) (Fig. 7H-J). The difference between the two groups was statistically significant, indicating that higher GCH1 expression was associated with lower CD163 expression and may protect against M2 macrophage infiltration, which can be used to explain why the higher the expression of GCH1 in the cancer tissue is, the better the prognosis in these patients with cervical cancer.

### 3.11 Relationships between GCH1 and the biological behavior of cervical cancer

**Figure 8:**
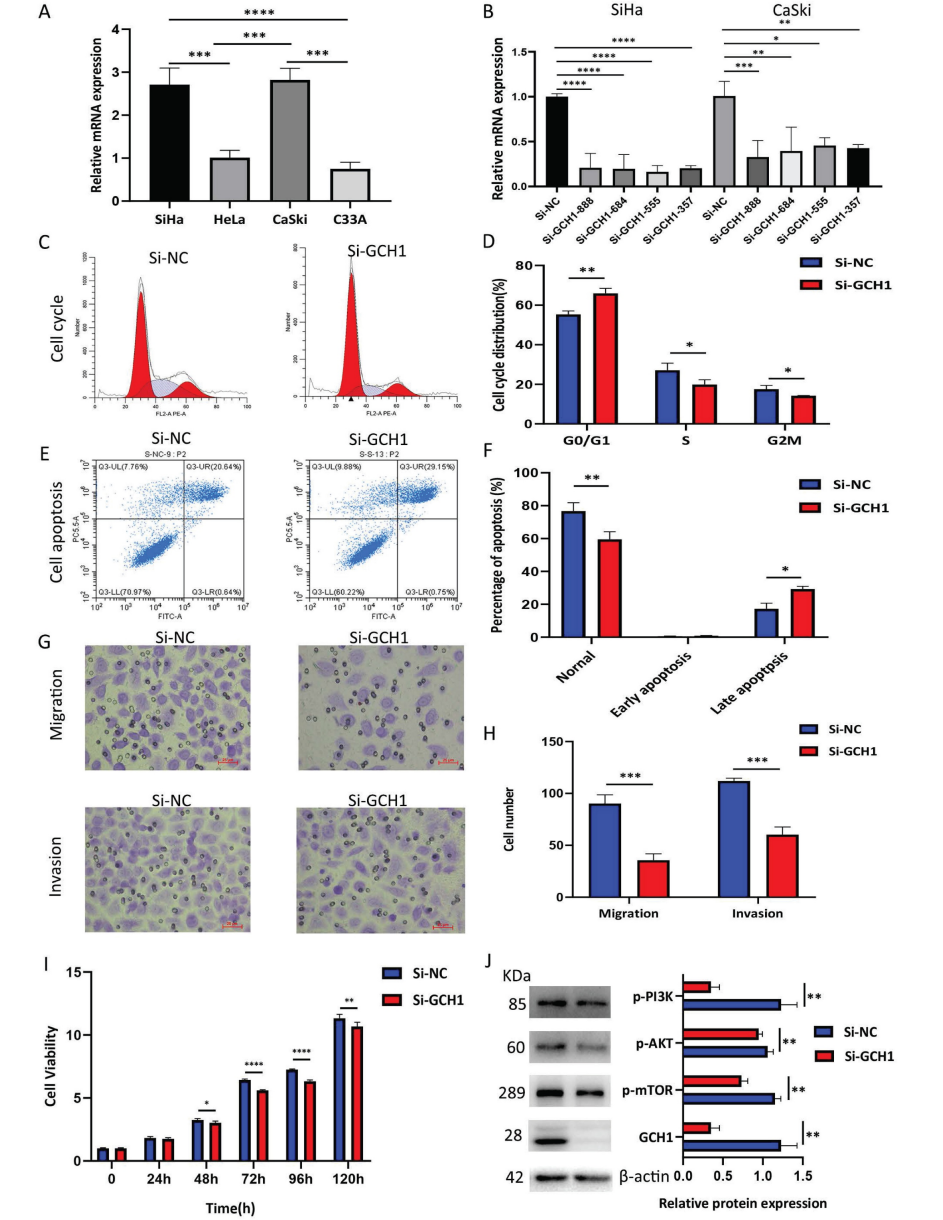
Relationship between GCH1 and the biological behavior of cervical cancer. (A) Relative mRNA expression levels of GCH1 in cervical cancer cell lines. (B) Relative mRNA expression levels of GCH1 in SiHa and CaSki cells transfected with si-GCH. (C-D) Cell cycle distribution of SiHa cells transfected with si-GCH1. (E-F) Apoptosis of SiHa cells transfected with si-GCH1. (G-H) Migration and invasion abilities of SiHa cells transfected with si-GCH1. (I) Viability of GCH1-knockdown SiHa cells, as determined by CCK-8 assay. (J) Expression levels of p-PI3K, p-AKT, p-mTOR, and GCH1 in SiHa knockdown cells. β-actin was used as an endogenous control. Each experiment was repeated at least 3 times. The data are presented as the means ± SEMs, *p < 0.05, **p < 0.01, ***p < 0.005, ****p < 0.001.

GCH1 expression was detected in cervical cancer cell lines using WB, and GCH1 was more highly expressed in SiHa and CaSki cells (Fig. 8A). The knockdown of GCH1 using siRNA was effective in both the SiHa and CaSki cell lines, with more pronounced effects observed in SiHa Si-GCH1-555 cells (Fig. 8B). Compared with the control, GCH1 knockdown induced significant G1 phase cell cycle arrest (p < 0.001) (Fig. 8C-D) and promoted apoptosis (p < 0.05) (Fig. 8E-F). Silencing GCH1 significantly inhibited both cell migration and invasion (p < 0.0001) (Fig. 8G-H) and reduced cell viability (Fig. 8I). Mechanistically, GCH1 silencing downregulated the PI3K/AKT/mTOR signaling pathway at the protein level (Fig. 8J). Collectively, these results demonstrate that GCH1 is associated with more aggressive phenotypes in cervical cancer, suggesting its oncogenic role in cervical cancer.

### 3.12 Effects of GCH1 in cancer cells on macrophage polarization

**Figure 9:**
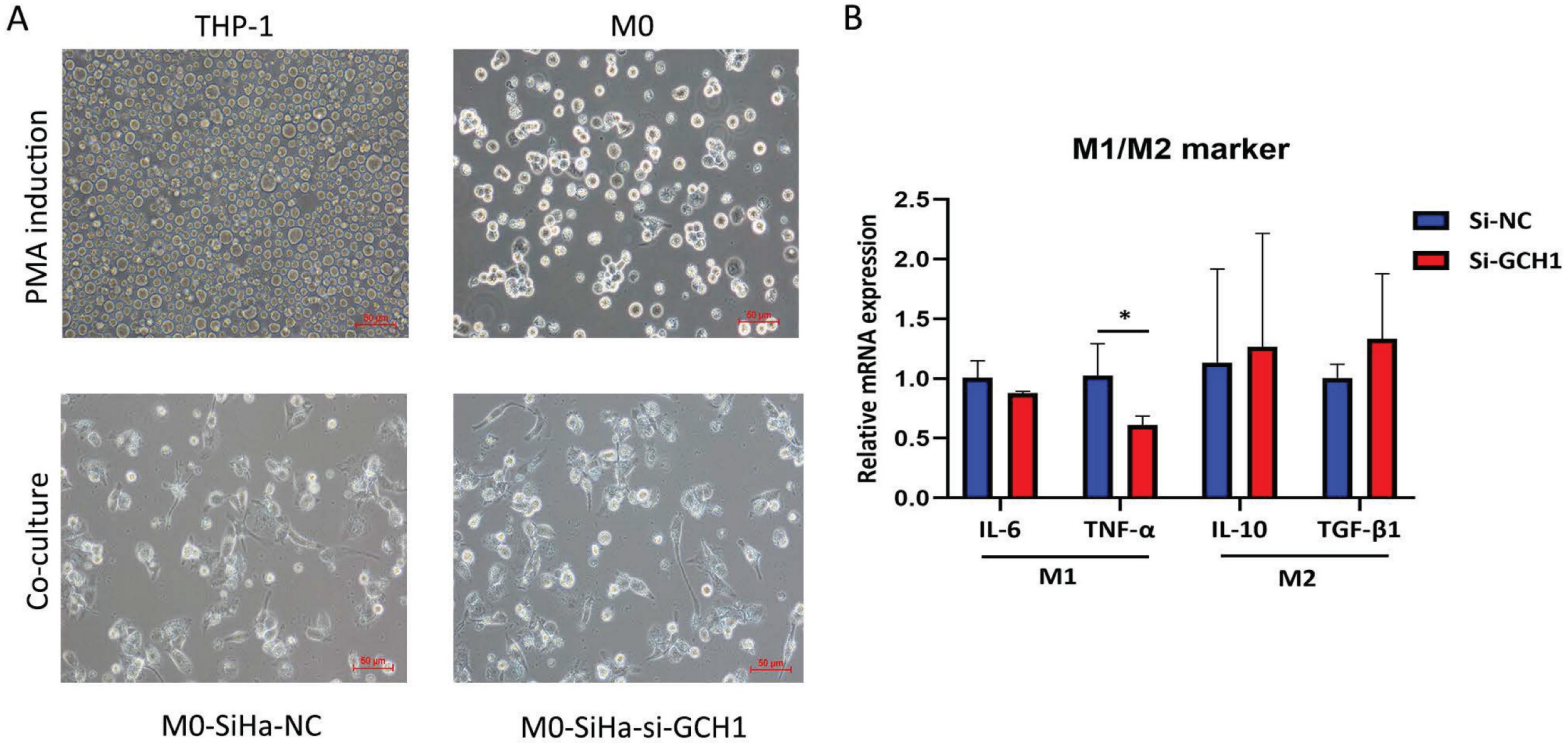
Effects of GCH1 in cancer cells on macrophage polarization. (A) Morphology of THP-1/M0 macrophages in the GCH1-knockdown and control groups. (B) Relative mRNA expression levels of M1 and M2 marker genes.

To study whether GCH1 in cervical cancer cells affects macrophage polarization, we collected conditioned medium (CM) from GCH1-knockdown SiHa cell cultures and cultured M0 cells with the CM. The M0 cells were subsequently analyzed. The utilization of CM from SiHa-si-GCH1 cells led to the downregulation of M1-marker genes, including IL-6 and TNF-α, whereas the expression levels of genes associated with an M2-like phenotype (i.e., IL-10 and TGF-β1) did not differ between M0 cells cultured with SiHa-si-NC CM and those cultured with SiHa-si-GCH1 CM (Fig. 9A-B), demonstrating that GCH1 in cervical cancer cells promoted M1 polarization in macrophages.

## 4. Discussion

Ferroptosis is often inhibited due to the increased antioxidant capacity in cancer cells and therefore has great potential for cancer treatment 5, 7, 19, 20. and further mechanistic studies have suggested that ferroptosis is regulated by numerous tumor-relevant genes and signaling networks 21. As a result, numerous factors influence whether a malignancy is more sensitive or resistant to ferroptosis induction, including its distinct genetic background and the surrounding immunological microenvironment 10. In cervical cancer, basic research results have suggested that the early expression of the viral proteins E1, E2, E6, and E7 from HPV types 16 and 18 is closely related to the generation of reactive oxygen species (ROS) and the concentration of reduced glutathione (GSH) 22; thus, we speculate that ferroptosis plays important roles in the occurrence and development of cervical cancer, and it is necessary to explore their relationships as well as key ferroptosis-related genes for treatment. The Kaplan‒Meier plot of ferroptosis-related gene scores from the TCGA database also provided clear evidence of the prognostic significance of ferroptosis-related gene sets in cervical cancer. Through the subsequent bioinformatics analysis, we discovered two effective ferroptosis-related molecules that have not yet been reported in cervical cancer: GCH1 and H1.2.

GCH1, also known as GTP cyclohydrolase-1, is the rate-limiting enzyme in tetrahydrobiopterin biosynthesis and is a significant antioxidant against ferroptosis 21. In humans, GCH1 is reported to be important for many diseases, including pain sensitivity, DOPA-responsive dystonia, hypertension, and Parkinson's disease 23-26. The role of GCH1 in cancer is highly controversial: GCH1 promotes the progression of gastric cancer, glioblastoma and triple-negative breast cancer by influencing proliferation and metastasis as well as the TME 27-29. In contrast, the silencing of GCH1 promotes hepatocellular carcinoma growth by inhibiting ASK1/p38 signaling 30, and its role in cervical cancer remains to be elucidated.

Our research revealed an interesting phenomenon: the expression of GCH1 in cervical cancer is greater than that in normal tissues, but the lower the expression in cervical cancer is, the better the prognosis. GCH1 knockdown impaired growth, migration and invasion and promoted apoptosis in cervical cancer cells, possibly through the inhibition of the phosphorylated PI3K/AKT/mTOR signaling pathway. This famous cancer-promoting signaling pathway in cervical cancer 31-33 was enriched in the GSVA analysis of GCH1. This could explain the elevated expression of GCH1, an oncogene, in cervical cancer tissues compared with normal tissues. In the TME of cervical cancer, GCH1 was positively linked with M1 macrophages and CD8+ T cells. These immune cells have significant anticancer activity and can release proinflammatory cytokines such as IL-6, IL-12, and IFN-γ 34-36. Furthermore, an examination of the interaction between GCH1 and the immunological checkpoint revealed that GCH1 was strongly negatively linked with CD276, which was previously identified as a carcinogenic molecule in cervical cancer in our studies 18, 37. Additionally, immunofluorescence spatial distance analysis revealed that reduced GCH1 expression could lead to increased CD163+ M2 macrophage infiltration. CD163+ M2 macrophages can aid in the immunological escape of tumor cells and increase tumor growth. The results of the cell coculture experiments also indicated that GCH1 knockdown in SiHa cells prevented the M1-like polarization of THP-1 cells after coculture. Moreover, increased expression of GCH1 enhances the immunogenicity of cancer cells; herein, knockdown of GCH1 leads to the disruption of immune-related anticancer responses. This could explain the better prognosis for patients with high expression of GCH1 in cervical cancer. These results revealed the dual effects of GCH1 in promoting cervical cancer cells and its anticancer effect on the immune microenvironment.

H1.2 is a member of the H1 linker histone family and is essential for the stability of higher-order structures in chromatin and nucleosomes. Numerous studies have shown that H1.2 plays important roles in a variety of disorders, including cancer, autoimmune diseases, and viral infections 38. It may enhance hepatocarcinogenesis in humans by influencing signal transducer and activator of transcription 3 (STAT3) signaling 39. Another study of B-cell lymphomas revealed that the H1 family, including H1.2, is a true tumor suppressor and that H1 mutations cause malignant transformation predominantly through three-dimensional genomic reorganization 40. In a cervical cancer investigation, immune infiltration and a necroptosis-related gene signature were reported to predict prognosis using bioinformatics analysis, although without verification 41. H1.2 expression was significantly higher in cervical cancer tissues than in normal tissues in the database at the mRNA level, but its expression in the TMA is still a matter of debate, as there were no significant results at the protein level, possibly because of the different techniques used to quantify H1.2 expression. We believe that H1.2 plays important roles in cervical cancer carcinogenesis because H1.2 directs the genome-wide chromatin association of the retinoblastoma tumor suppressor protein (pRb) and facilitates its function 42. Furthermore, H1.2 has the potential to inhibit the transcription of the p16 tumor suppressor gene 43. pRb and p16 are important tumor suppressor genes in cervical cancer 44-46, but more laboratory evidence is needed to support this theory.

Although ferroptosis is distinct from other forms of programmed cell death, such as apoptosis, autophagy, necroptosis, and pyroptosis, these pathways can interconvert and form a complex network under different stress conditions 12, 47, 48. Thus, it is speculated that GCH1 and H1.2 may not be limited to ferroptosis but could also play roles in other forms of programmed cell death, necessitating further experimental research to clarify their interaction and its mechanisms.

In conclusion, we conducted a systematic examination of the ferroptosis genomic expression profile in cervical cancer and discovered two significant ferroptosis-related biomarkers. Moreover, we identified the complexity of the function of GCH1 in cervical cancer. However, more laboratory work is needed to explore their roles and mechanisms in tumorigenesis and the TME, including *in vivo* experiments, to verify their efficacy as therapeutic targets.

## 5. Limitations of the study

First, *in vivo* animal experiments were not conducted, and GCH1 blockade combined with immunotherapy may be an effective treatment approach for cervical cancer. Second, the molecular mechanisms related to the polarization of macrophages to the M1 phenotype after cell coculture still need to be further explored.

## Supplementary Material

Supplementary figures and tables.

## Figures and Tables

**Table 1:** Association between GCH1 expression and clinicopathological factors.

Feature	Patients	GCH1		χ^2^	*p* value
		Low 34	High 88		
Age					
<45	52	15	37	0.04306	0.8356
≥45	70	19	51		
HPV					
Positive	95	26	69	0.003216	0.9548
Negative	15	4	11		
Lymph node metastasis					
Present	25	11	14	2.107	0.1456
Absent	97	23	74		
Histological grade					
I	20	4	16	4.297	0.1166
II	13	7	6		
III	89	23	66		
FIGO stage					
I	68	11	57	10.51	**0.01047**
II	28	12	16		
III	24	10	14		
IV	2	1	1		
Recurrence					
Present	39	15	24	3.200	0.0736
Absent	83	19	64		

## References

[1] Bray F, Laversanne M, Sung H, Ferlay J, Siegel RL, Soerjomataram I (2024). Global cancer statistics 2022: GLOBOCAN estimates of incidence and mortality worldwide for 36 cancers in 185 countries. CA: a cancer journal for clinicians.

[2] Han B, Zheng R, Zeng H, Wang S, Sun K, Chen R (2024). Cancer incidence and mortality in China, 2022. Journal of the National Cancer Center.

[3] Lee YJ, Kim DY, Lee SW, Park JY, Suh DS, Kim JH (2017). A postoperative scoring system for distant recurrence in node-positive cervical cancer patients after radical hysterectomy and pelvic lymph node dissection with para-aortic lymph node sampling or dissection. Gynecologic oncology.

[4] Tewari KS, Sill MW, Penson RT, Huang H, Ramondetta LM, Landrum LM (2017). Bevacizumab for advanced cervical cancer: final overall survival and adverse event analysis of a randomised, controlled, open-label, phase 3 trial (Gynecologic Oncology Group 240). Lancet (London, England).

[5] Tossetta G, Marzioni D (2023). Targeting the NRF2/KEAP1 pathway in cervical and endometrial cancers. European journal of pharmacology.

[6] Lei G, Zhuang L, Gan B (2024). The roles of ferroptosis in cancer: Tumor suppression, tumor microenvironment, and therapeutic interventions. Cancer cell.

[7] Fantone S, Piani F, Olivieri F, Rippo MR, Sirico A, Di Simone N (2024). Role of SLC7A11/xCT in Ovarian Cancer. International journal of molecular sciences.

[8] Dixon SJ, Lemberg KM, Lamprecht MR, Skouta R, Zaitsev EM, Gleason CE (2012). Ferroptosis: an iron-dependent form of nonapoptotic cell death. Cell.

[9] Stockwell BR (2022). Ferroptosis turns 10: Emerging mechanisms, physiological functions, and therapeutic applications. Cell.

[10] Jiang X, Stockwell BR, Conrad M (2021). Ferroptosis: mechanisms, biology and role in disease. Nature reviews Molecular cell biology.

[11] Wang T, Gong M, Cao Y, Zhao C, Lu Y, Zhou Y (2022). Persistent ferroptosis promotes cervical squamous intraepithelial lesion development and oncogenesis by regulating KRAS expression in patients with high risk-HPV infection. Cell death discovery.

[12] Samare-Najaf M, Samareh A, Savardashtaki A, Khajehyar N, Tajbakhsh A, Vakili S (2024). Non-apoptotic cell death programs in cervical cancer with an emphasis on ferroptosis. Critical reviews in oncology/hematology.

[13] Gong M, Shen F, Li Y, Ming L, Hong L (2023). MLK4 as an immune marker and its correlation with immune infiltration in Cervical squamous cell carcinoma and endocervical adenocarcinoma(CESC). PloS one.

[14] Chen W, Chen X, Wang Y, Liu T, Liang Y, Xiao Y (2019). Construction and Analysis of lncRNA-Mediated ceRNA Network in Cervical Squamous Cell Carcinoma by Weighted Gene Co-Expression Network Analysis. Medical science monitor: international medical journal of experimental and clinical research.

[15] Xu X, Liu T, Wu J, Wang Y, Hong Y, Zhou H (2019). Transferrin receptor-involved HIF-1 signaling pathway in cervical cancer. Cancer gene therapy.

[16] Song J, Ye A, Jiang E, Yin X, Chen Z, Bai G (2018). Reconstruction and analysis of the aberrant lncRNA-miRNA-mRNA network based on competitive endogenous RNA in CESC. Journal of cellular biochemistry.

[17] Li L, Guo Q, Lan G, Liu F, Wang W, Lv X (2022). Construction of a four-mRNA prognostic signature with its ceRNA network in CESC. Scientific reports.

[18] Han S, Wang Y, Shi X, Zong L, Liu L, Zhang J (2018). Negative roles of B7-H3 and B7-H4 in the microenvironment of cervical cancer. Experimental cell research.

[19] Yang J, Ma S, Xu R, Wei Y, Zhang J, Zuo T (2021). Smart biomimetic metal organic frameworks based on ROS-ferroptosis-glycolysis regulation for enhanced tumor chemo-immunotherapy. Journal of controlled release: official journal of the Controlled Release Society.

[20] Liu Z, Kang R, Yang N, Pan X, Yang J, Yu H (2024). Tetrahydrobiopterin inhibitor-based antioxidant metabolic strategy for enhanced cancer ferroptosis-immunotherapy. Journal of colloid and interface science.

[21] Ding X, Cui L, Mi Y, Hu J, Cai Z, Tang Q (2025). Ferroptosis in cancer: revealing the multifaceted functions of mitochondria. Cellular and molecular life sciences: CMLS.

[22] Cruz-Gregorio A, Manzo-Merino J, Gonzaléz-García MC, Pedraza-Chaverri J, Medina-Campos ON, Valverde M (2018). Human Papillomavirus Types 16 and 18 Early-expressed Proteins Differentially Modulate the Cellular Redox State and DNA Damage. International journal of biological sciences.

[23] Campbell CM, Edwards RR, Carmona C, Uhart M, Wand G, Carteret A (2009). Polymorphisms in the GTP cyclohydrolase gene (GCH1) are associated with ratings of capsaicin pain. Pain.

[24] Weissbach A, Pauly MG, Herzog R, Hahn L, Halmans S, Hamami F (2022). Relationship of Genotype, Phenotype, and Treatment in Dopa-Responsive Dystonia: MDSGene Review. Movement disorders: official journal of the Movement Disorder Society.

[25] Dickinson Y, Boehni R, Obeid R, Knapp JP, Moser R, Lewandowski AJ (2024). Novel Role of 5-Methyl-(6S)-Tetrahydrofolate in Mediating Endothelial Cell Tetrahydrobiopterin in Pregnancy and Implications for Gestational Hypertension. Hypertension (Dallas, Tex: 1979).

[26] Pan HX, Zhao YW, Mei JP, Fang ZH, Wang Y, Zhou X (2020). GCH1 variants contribute to the risk and earlier age-at-onset of Parkinson's disease: a two-cohort case-control study. Translational neurodegeneration.

[27] Liu Y, Zhai E, Chen J, Qian Y, Zhao R, Ma Y (2022). m(6) A-mediated regulation of PBX1-GCH1 axis promotes gastric cancer proliferation and metastasis by elevating tetrahydrobiopterin levels. Cancer communications (London, England).

[28] Jiang Y, Zhao J, Li R, Liu Y, Zhou L, Wang C (2022). CircLRFN5 inhibits the progression of glioblastoma via PRRX2/GCH1 mediated ferroptosis. Journal of experimental & clinical cancer research: CR.

[29] Wei JL, Wu SY, Yang YS, Xiao Y, Jin X, Xu XE (2021). GCH1 induces immunosuppression through metabolic reprogramming and IDO1 upregulation in triple-negative breast cancer. Journal for immunotherapy of cancer.

[30] Zhong GC, Zhao ZB, Cheng Y, Wang YB, Qiu C, Mao LH (2021). Epigenetic silencing of GCH1promotes hepatocellular carcinoma growth by activating superoxide anion-mediated ASK1/p38 signaling via inhibiting tetrahydrobiopterin *de novo* biosynthesis. Free radical biology & medicine.

[31] Zhang L, Wu J, Ling MT, Zhao L, Zhao KN (2015). The role of the PI3K/Akt/mTOR signalling pathway in human cancers induced by infection with human papillomaviruses. Molecular cancer.

[32] Husseinzadeh N, Husseinzadeh HD (2014). mTOR inhibitors and their clinical application in cervical, endometrial and ovarian cancers: a critical review. Gynecologic oncology.

[33] Skelin J, Luk HY, Butorac D, Boon SS, Tomaić V (2023). The effects of HPV oncoproteins on host communication networks: Therapeutic connotations. Journal of medical virology.

[34] Gunassekaran GR, Poongkavithai Vadevoo SM, Baek MC, Lee B (2021). M1 macrophage exosomes engineered to foster M1 polarization and target the IL-4 receptor inhibit tumor growth by reprogramming tumor-associated macrophages into M1-like macrophages. Biomaterials.

[35] Cui C, Wang J, Fagerberg E, Chen PM, Connolly KA, Damo M (2021). Neoantigen-driven B cell and CD4 T follicular helper cell collaboration promotes anti-tumor CD8 T cell responses. Cell.

[36] Ma S, Sun B, Duan S, Han J, Barr T, Zhang J (2023). YTHDF2 orchestrates tumor-associated macrophage reprogramming and controls antitumor immunity through CD8(+) T cells. Nature immunology.

[37] Han S, Shi X, Liu L, Zong L, Zhang J, Chen Q (2018). Roles of B7-H3 in Cervical Cancer and Its Prognostic Value. Journal of Cancer.

[38] Kim JM, Kim K, Punj V, Liang G, Ulmer TS, Lu W (2015). Linker histone H1.2 establishes chromatin compaction and gene silencing through recognition of H3K27me3. Scientific reports.

[39] Wang Q, Chen Y, Xie Y, Yang D, Sun Y, Yuan Y (2022). Histone H1.2 promotes hepatocarcinogenesis by regulating signal transducer and activator of transcription 3 signaling. Cancer science.

[40] Yusufova N, Kloetgen A, Teater M, Osunsade A, Camarillo JM, Chin CR (2021). Histone H1 loss drives lymphoma by disrupting 3D chromatin architecture. Nature.

[41] Xing X, Tian Y, Jin X (2022). Immune infiltration and a necroptosis-related gene signature for predicting the prognosis of patients with cervical cancer. Frontiers in genetics.

[42] Munro S, Hookway ES, Floderer M, Carr SM, Konietzny R, Kessler BM (2017). Linker Histone H1.2 Directs Genome-wide Chromatin Association of the Retinoblastoma Tumor Suppressor Protein and Facilitates Its Function. Cell reports.

[43] Kotake Y, Tanigawa Y, Tarumi R (2023). Histone H1.2 Represses the Transcription of the p16 Tumor Suppressor Gene. Anticancer research.

[44] Craig C, Kim M, Ohri E, Wersto R, Katayose D, Li Z (1998). Effects of adenovirus-mediated p16INK4A expression on cell cycle arrest are determined by endogenous p16 and Rb status in human cancer cells. Oncogene.

[45] Zhou J, Li B, Peng C, Wang F, Fu Z, Zhou C (2013). Inhibition of cervical cancer cell growth *in vitro* and *in vivo* by lentiviral-vector mediated shRNA targeting the common promoter of HPV16 E6 and E7 oncogenes. Antiviral research.

[46] Tringler B, Gup CJ, Singh M, Groshong S, Shroyer AL, Heinz DE (2004). Evaluation of p16INK4a and pRb expression in cervical squamous and glandular neoplasia. Human pathology.

[47] Chang X, Miao J (2023). Ferroptosis: Mechanism and potential applications in cervical cancer. Frontiers in molecular biosciences.

[48] Li P, Lv X, Liu L, Peng M, Qin D (2022). The Role of Ferroptosis-Related Molecules and Significance of Ferroptosis Score in Cervical Cancer. Journal of oncology.

