# Supplementary material for: Discovering the abnormalities and functional importance of ferroptosis-related molecules in cervical cancer
Source: Int J Med Sci. 2026 Jan 1;23(2):443–60. doi: 10.7150/ijms.107133 (PMC12825119; doi:10.7150/ijms.107133)
Supplement: Supplementary file 1 — Supplementary figures and tables. [file ijmsv23p0443s1.zip › Supplementary materials/Supplementary Figures and Table S3.pdf]

Supplementary Figure 1

A

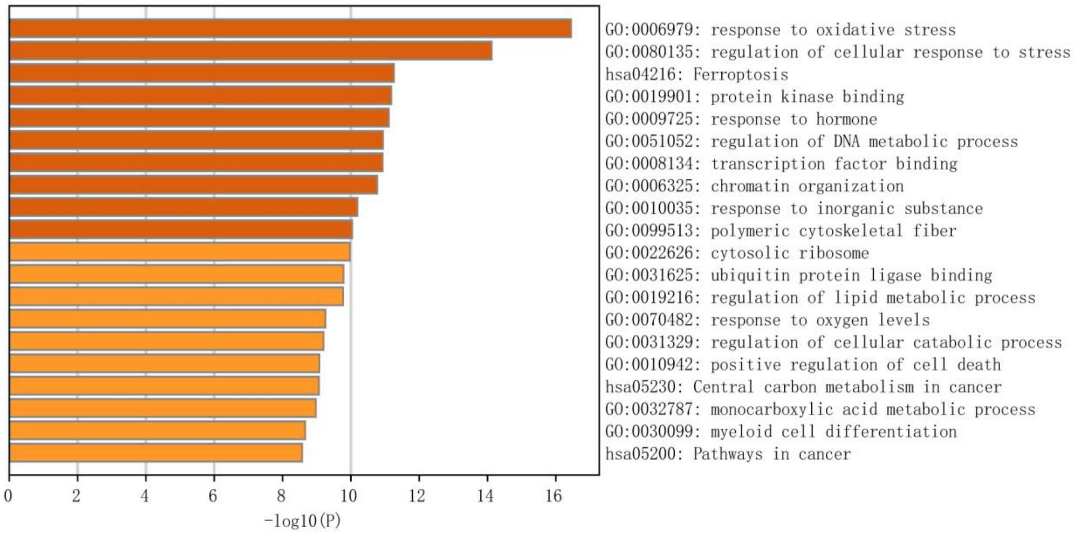

B

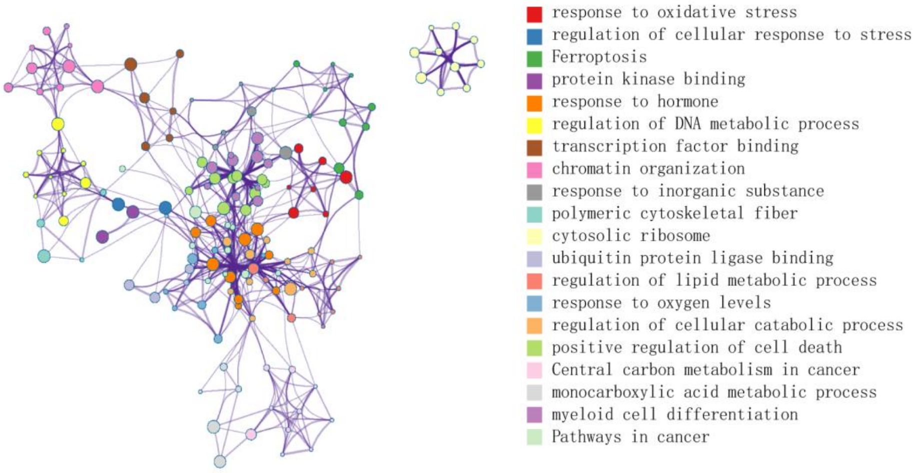

Supplementary Figure 2

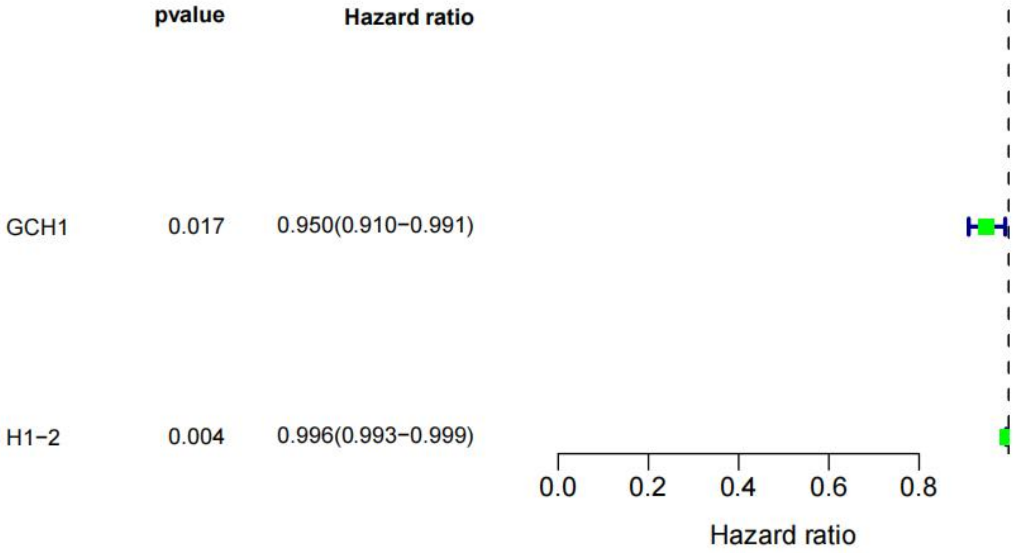

Supplementary Figure 3

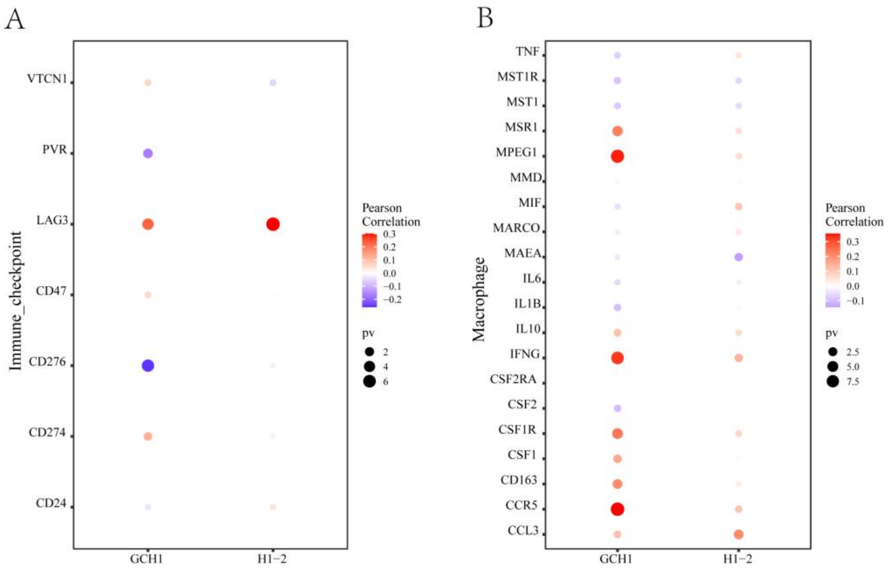

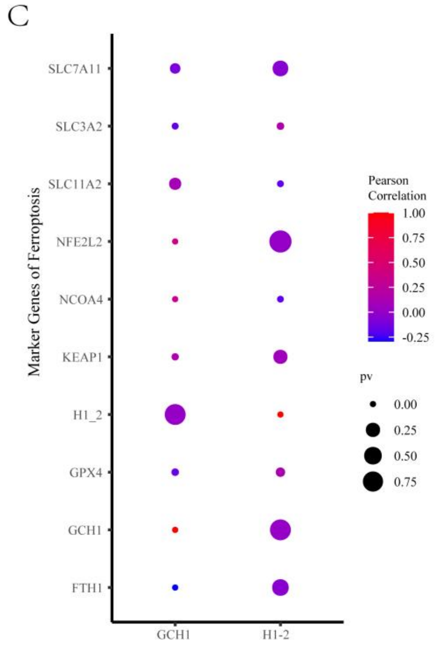

Supplementary Figure 4

A

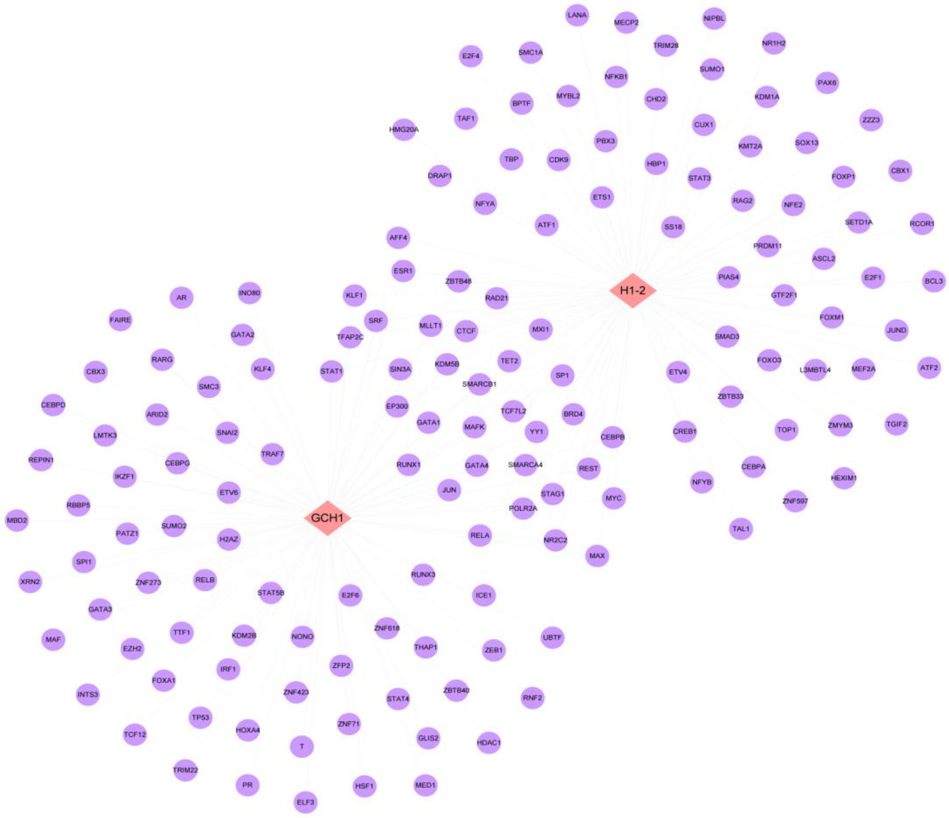

B

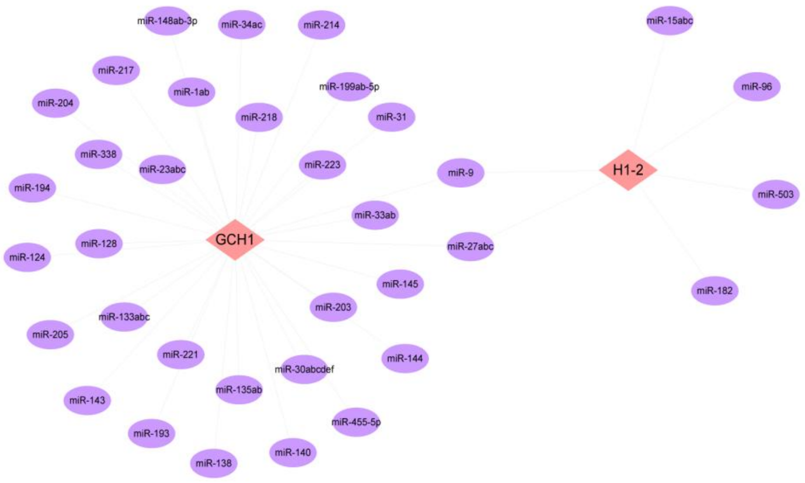

Supplementary Figure 5

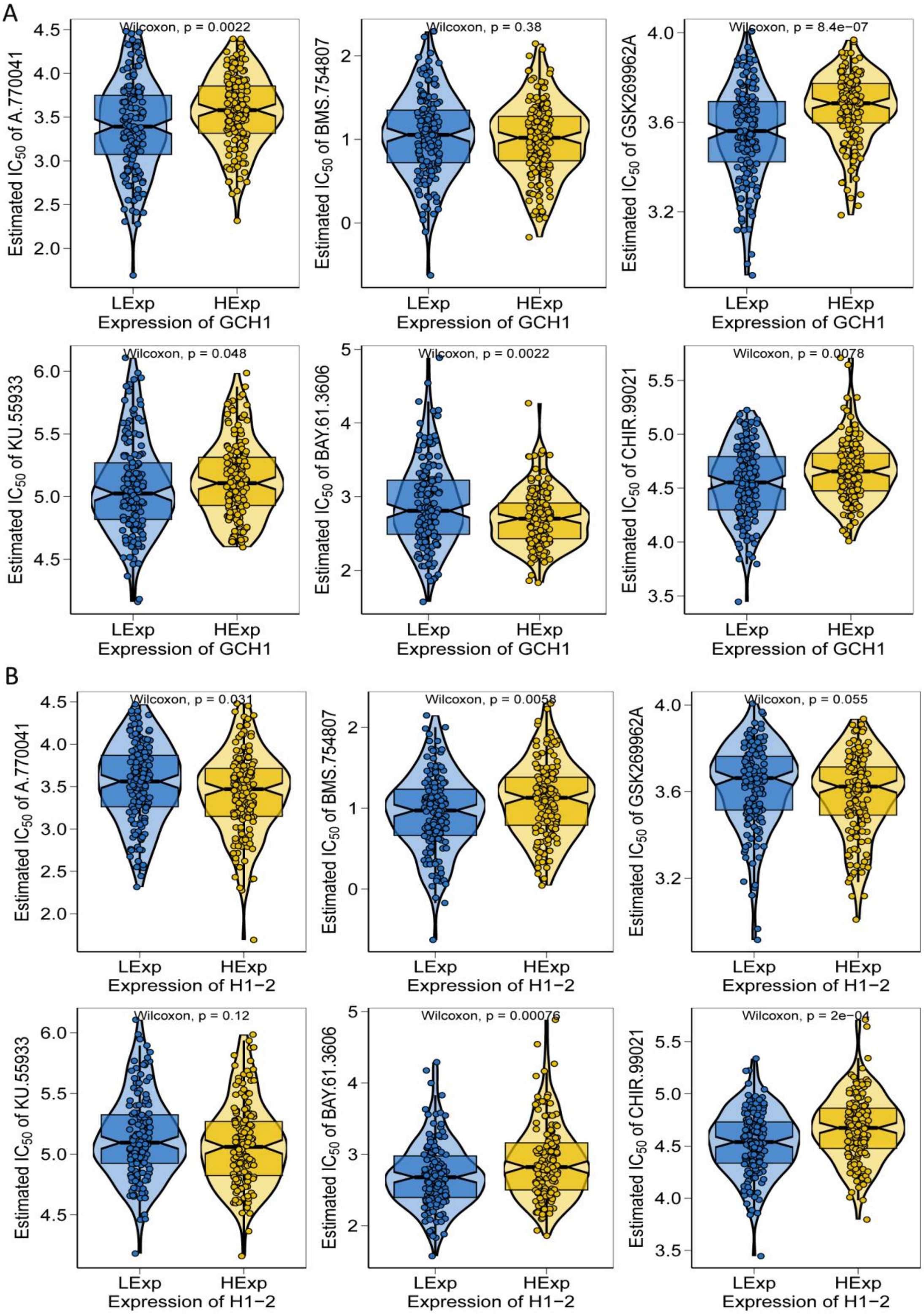

Supplementary Figure 6

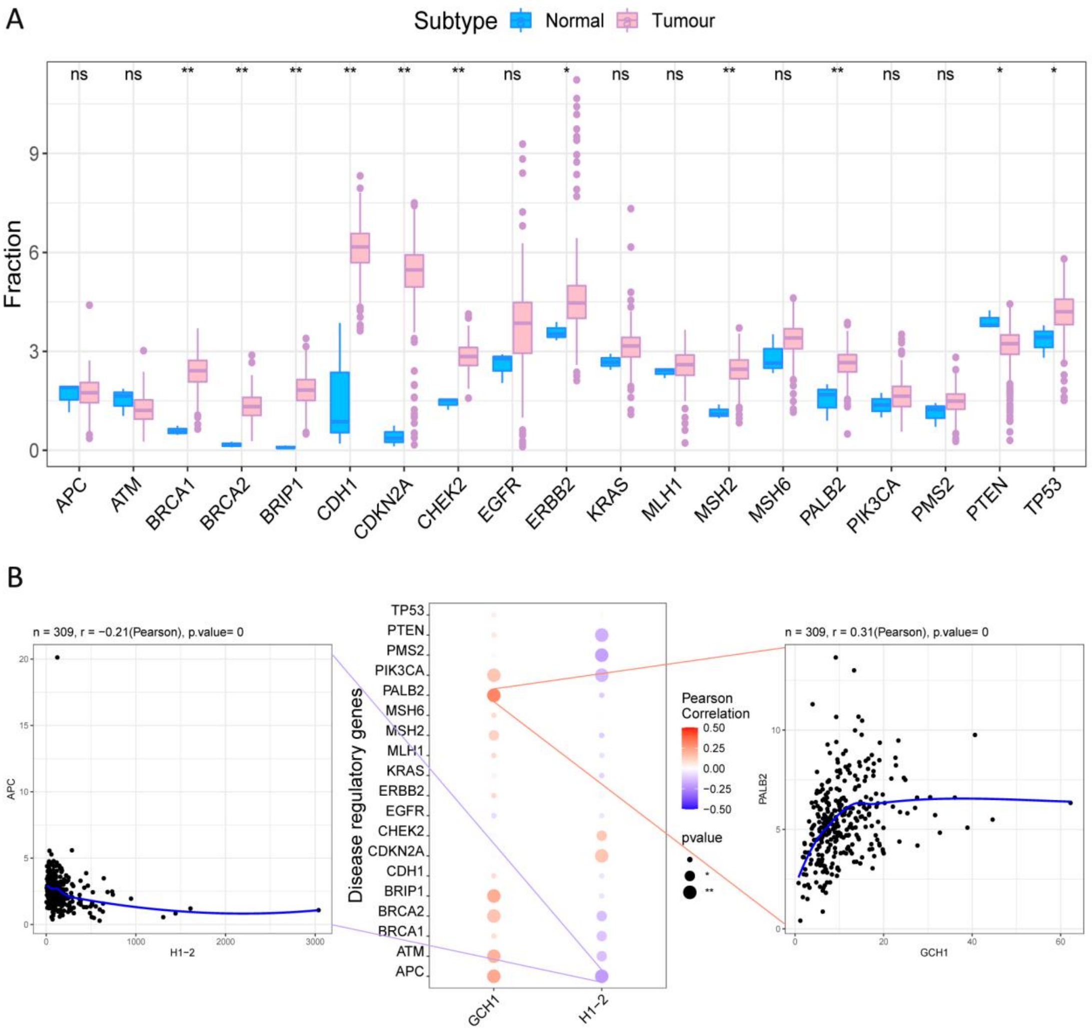

Supplementary Figure 7

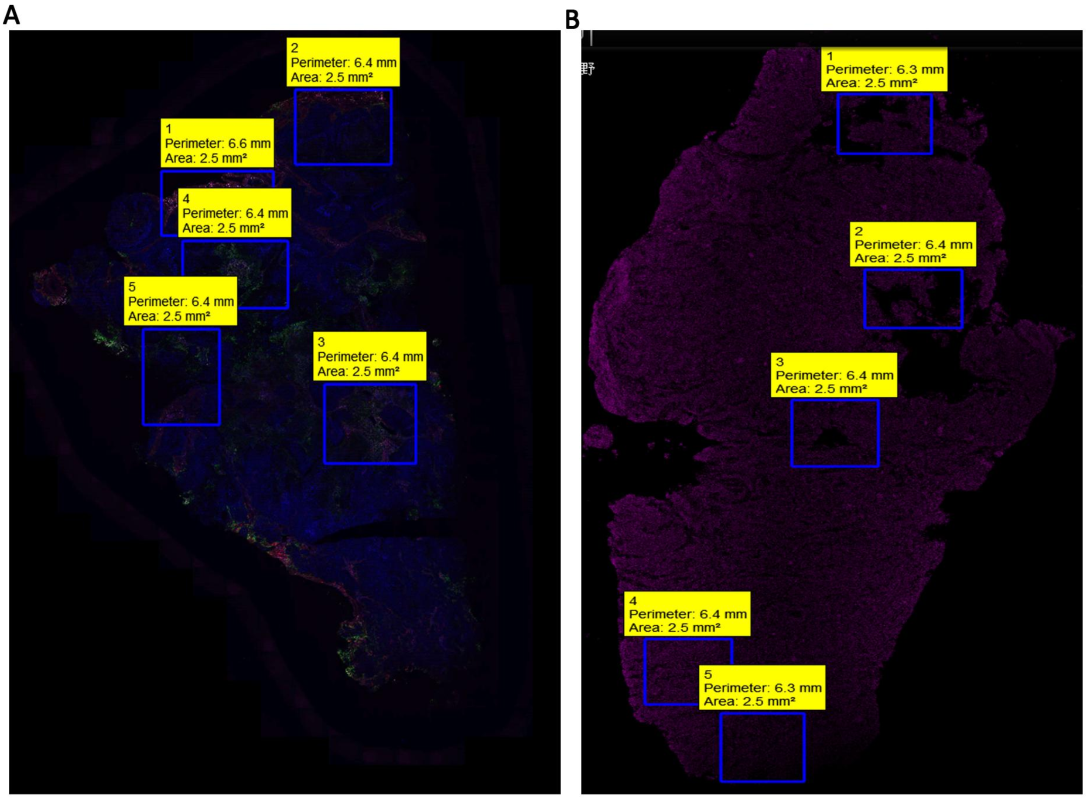

Supplymentary Table S3. Association between H1.2 expression and clinicopathological factors.

Feature	Patients	H1.2		χ^2	*p*-value
		Low	High		
Age					
<45	52	34	18	8.176	0.0042
≥45	69	27	42		
HPV					
Positive	94	49	45	0.1544	0.6943
Negative	15	7	8		
Lymph node metastasis					
Present	24	15	9	1.750	0.1859
Absent	97	46	51		
Histological grade					
I	20	7	13	2.666	0.1859
II	13	8	5		
III	88	46	42		
FIGO stage					
I	68	32	36	4.358	0.2253
II	28	14	14		
III	23	15	8		
IV	2	0	2		
Recurrence					
Present	39	18	21	0.4177	0.5181
Absent	82	43	39		
